# Ethnobotanical Study of Herbaceous Flora along an Altitudinal Gradient in Bharmour Forest Division, District Chamba of Himachal Pradesh, India

**DOI:** 10.1155/2014/946870

**Published:** 2014-04-27

**Authors:** Kehar S. Thakur, Munesh Kumar, Rajan Bawa, Rainer W. Bussmann

**Affiliations:** ^1^College of Forestry, Dr. Y.S. Parmar University of Horticulture and Forestry, Nauni, Solan, Himachal Pradesh 173 230, India; ^2^Department of Forestry and Natural Resources, H.N.B. Garhwal University, Srinagar, Garhwal, Uttarakhand 249161, India; ^3^William L. Brown Center, Missouri Botanical Garden, St. Louis, MO 63110, USA

## Abstract

The present ethnobotanical study was carried out in Holi (Deol, Kut, Dal, and Lahaud Dhar) forest range and in Bharmor (Seri, Bharmour, Malkauta, Bharmani, Harsar, Dhancho, Sundrasi, Gorikund, and Manimahesh) forest range to obtain information on the plants used by the local inhabitants for several purposes. A total of 54 plants were recorded in this study. The plants are employed to treat simple diseases (cough, cold, fever, and burns) and some serious diseases (typhoid, jaundice, and kidney disease). Some of the plants are also used as incense for religious ceremonies and several other daily needs. But due to absence of scientific monitoring of plants, their cultivation, harvesting, and management techniques as well as sustainable use and lack of awareness of social factors, the availability of valuable plant resources is decreasing at an alarming rate. In addition, the indigenous knowledge regarding the use of lesser-known plants of this region is also rapidly declining. Therefore, the documentation of plant resources is a necessary step towards the goal of raising awareness in local communities about the importance of these plants and their further conservation.

## 1. Introduction

Ethnobotany is widely regarded as the science of human interaction with plants and their environments. Ethnobotanical knowledge is the result of successful experimentation with plants since time immemorial and has given us our recognized foods and medicines. Ethnobotany illuminates the direct relationship between human beings and plants and has proven to be of great utility in the health care programs. Ethnobotany also explores the importance of plants as emergency foods, as well as uncovering useful information about the sociocultural medicoreligious lore and values, phrases and proverbs, taboos, and totems prevailing in a specific region or society. Over the last century, ethnobotany has evolved into a scientific discipline that focuses on the people and plant relationship in a multidisciplinary manner, incorporating not only collection and documentation of indigenously used species but also ecology, economy, pharmacology, public health, and other disciplines.

Today, ethnobotany has become increasingly valuable in the development of health care and conservation programs in different parts of the world. Ethnobotanical studies that explore and help to preserve knowledge are therefore urgently needed before traditional folklores are lost forever [1]. The dependence on herbal resources to cure different types of diseases is well known. It has been estimated that there are between 3,500 and 70,000 plant species that have been used around the world, at one time or another, for medicinal purpose. At least 65,000 species are used in Asia alone as home remedies for various ailments [2]. The World Health Organization (WHO) has estimated that at least 80 percent of the world's population relies on traditional systems of medicine to meet their primary health care needs. In addition, medicinal plants also form an important part of the world's economy since many modern medicines are derived from plants. The indigenous systems of medicine practiced in India are mainly based on the use of plants. Every year, the medicinal plant-related trade is growing rapidly, and while India's share in global market is not very impressive (only 0.5%–1%), demand for these products is increasing at an alarming rate [3].

The rural and tribal people of India still depend largely on the local herbal resources for curing different types of diseases. The use of plants as medicine dates back to the early man. There are records of the use of medicinal plants by ancient great civilizations, such as those of China, India, the Middle East, North Africa, and South America. This culture continues today in the form of folk medicine in different parts of the world and led to the development of traditional systems of medicine. Systematic and scientific investigations of traditional medicinal plants have also provided many valuable drugs in western medicine [4].

The Chamba district of Himachal Pradesh is considered as one of the richest areas of traditional and potential medicinal wealth. The Kangra district of Himachal Pradesh and the Gurdaspur district of Punjab bound the district to the south, Jammu and Kashmir to the north, and Lahaul-Spiti to the east. The district has two tribal regions, namely, Pangi and Bharmour. Bharmour is situated in the west of this district, whereas the Pangi Valley is situated in the north. The vegetation of the Chamba district varies considerably, chiefly owing to elevation and rainfall variations [5]. There is no proper record available regarding the traditional medicinal knowledge of the tribal area except the study carried out by Rani et al. [6] from Chamba district of Himalachal Pradesh, which is a very limited study from this region. Keeping these factors in view, the present study was carried out with the objective to find out the various uses of the herbaceous flora used by the inhabitants in this region of Himachal Pradesh, India.

## 2. Materials and Methods

### 2.1. Study Area and Climate

**Figure 1:**
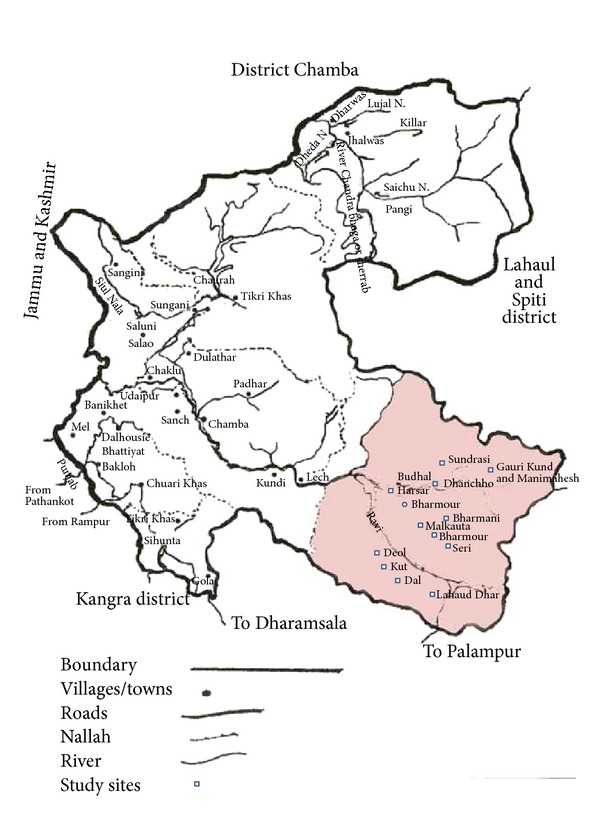


An extensive field survey of selected areas of Holi and Bharmour was carried out. Sites included Deol (2,300–2800 m), Kut (2,800–3300 m), Dal (3,300–3800 m), and Lahaud Dhar (3,800 m and above) in the Holi forest range and Seri (1,700–2200 m), Bharmour (2,250 m), Malkauta (2,550 m), Bharmani (2,900 m), Harsar (2,450 m), Dhanchho (2,800–3300 m), Sundrasi (3,300–3800 m), Gorikund, and Manimahesh (3,800 m and above) in the Bharmour forest range (Figure 1: location map of the study area).

The climate of the study area is typically temperate. The year is characterized by three main seasons: the cool and relatively dry winter (December to March), the warm and dry summer (mid-April to June), and a warm and wet period (July to mid-September), called the monsoon or rainy season. The rainy season accounts for about three quarters of the annual rainfall. Apart from these main seasons, the transitional periods connecting the rainy season and winter and winter and summer are referred to as autumn (October to November) and spring (February to March). The mean annual rainfall is 1500 mm, and the mean annual temperature lies between 3°C and 30°C.

### 2.2. Methodology Adopted

The information regarding the traditional knowledge, local uses of plants within the study area, the local names, parts used, purposes, modes of administration, and curative properties, and so forth was recorded through intensive interviews and discussions with elderly people (men/women), herbal healers, local vaids, and grazers (Gaddis and Gujjars) using a well- structured questionnaire (Annexure-1). The information on plants was collected randomly from approximate 10% of the total population (30 adult persons in Holi (Deol, Kut, Dal, and Lahaud Dhar) forest range and 20 adult persons in Bharmor range (Seri, Bharmour, Malkauta, Bharmani, Harsar, Dhancho, Sundrasi, Gorikund, and Manimahesh)). The information was taken from all ages. We tried to achieve an even age/gender distribution in all age classes. All information was obtained after receiving an oral prior informed consent from the participants, and the ISE (International Society of Ethnobiology) Code of Ethics was followed. The inhabitants identified the plants used for various purposes, and vouchers of each plant were collected and stored in the herbarium of the Department of Forest Products, Y.S. Parmar University, Solan, Himachal Pradesh. The HERBARIUM ACRONYM is given as UHF with collector number (Table 1). All scientific plant names follow TROPICOS (www.TROPICOS.org), and the nomenclature follows APG-3. In addition, we reviewed information on ethnobotanical uses mentioned in India's vast literature, as well as in related written sources, for example, [7, 8]. An oral consensus survey was also carried out among the people of each locality.

*Annexure-1.* Questionnaire used to collect information on plant use.

Informant Details

 Name:
 Sex:
 Age:
 Village:        Panchayat:
 Block:        District:
 Main occupation:    Subsidiary occupation:
 Education:

Ethnobotanical uses of plants.

1. Local/vernacular name of plant:
2. Scientific name of plant:
3. Part used of plant:
4. Name of ailment/other purposes in which plant part is used:
5. Mode of preparation:
6. Use (externally/internally):
7. Availability in natural habitat:
8. Cause of declining of ethnobotanical plants if any (overgrazing, encroachments, forest fire, mining activities, climatic change, and others):
9. Who knows best about plant and uses: vaids, shepherds, old people/new generation, and others:
10. Any ethnobotanical plant species under cultivation:
11. Any awareness camps /trainings /exposure visits organized for ethnobotanical plants:
12. Any conservation practices on ethnobotanical plants:

## 3. Results and Discussion

The ethnobotanical information about the various plants was collected through interviews and discussions with elderly/experienced people. The data reveal that villagers used 54 species for common ailments and other purposes (Table 1).

Local elderly people, hermits, shepherds, and vaids provided the information about different plant uses. Many of the plant species are used frequently (though sometimes only occasionally) for curing various diseases. The local people (shepherds in particular) believe in the healing power of these herbs, along with the power of Tantra and Mantra, but knowledge thereof is restricted to very few elderly folks. Moreover, the younger generation does not seem much interested in keeping this traditional knowledge alive and spends most of the time growing commercial crops and fruits. With the passage of time, knowledge about these valuable medicinal plant resources will vanish. In the future, the information will be completely lost, thereby greatly weakening traditional medical practices. Therefore, this valuable information needs to be systematically collected and documented, so that it can serve mankind for generations to come and may also conserve the precious plant resources of high economic utility.

The present study calls attention to some species with ethnobotanical uses that have not been reported earlier [9]. Although, the ethnobotanical study carried out by Sharma [10] of the Gaddi tribe of the Kangra district of Himachal Pradesh, where he documented 67 plants of ethnobotanical uses. Of those, some species recur in this study. However, there are certain variations in the ethnobotanical use of these plants. For example,* Origanum vulgare* was reported to have the properties of an insect repellent. We found that people in the Bharmour area use it instead to wash milk utensils in order to impart aroma to the milk. Similarly, Sharma [10] reported the use of* Angelica glauca *roots in case of dyspepsia; however, the present study reveals its use in treating flatulence and curing edema including dyspepsia. The difference in ethnobotanical practice may be due to the fact that the Gaddis have settled in Kangra for a very long time, during which they developed some different ways of utilizing plants.

Of the plants considered to have ethnobotanical uses recorded in the present study, some of them have been mentioned in the study conducted by Dinanath [4] and Gupta [9]. Many of these plants have almost the same ethnobotanical uses. However, there are slight variations. For instance, Dinanath [4] reported the use of* Angelica glauca *as flavoring agent and Gupta [9] reported this plant was useful for reducing obesity; however, Bhat et al. [11] conducted a study in Garhwal Himalayan forests which reported that* Angelica glauca* is used for indigestion and constipation, whereas we found that this species has many uses such as being used as a spice, treating flatulence and dyspepsia, and curing edema. Bhat et al. [11] also reported that* Picrorhiza kurroa *root is used for fever and stomachache; however, in the present study,* Picrorhiza kurroa *root is used for jaundice and diarrhea including stomachache; further, earlier studies describe* Heracleum candicans *as useful for healing of wounds, and the paste of the root is applied to counteract snake bite. In our interviews, we found* H. candicans* paste is useful in case of snake bite, including this, the paste also mixed with sour lassi and given to the patient. These differences in the ethnobotanical practice may be due to the variation in the place of study and objectives of studies, the former being carried out among the Pangi of the Pangwal tribe and the Gaddi tribe of Bharmour, whereas the present study reported anthropogenic pressure, along with ethnobotanical data found in the Bharmour forest division. Rani et al. [6] conducted a study on ethnomedicinal plants of Chamba district, Himachal Pradesh, which reported 50 plant species commonly used by local people to cure 26 diseases. Of total 50 plants reported by Rani et al. [6] in their study, some of them were commonly reported in the present study but they vary their mode of use and purposes. Kumar et al. [12] carried out a study on ethnomedicinal plants of Garhwal Himalaya where few plants were common in the present study but the uses were also reported differently. Bhat et al. [13] collected information on ethnomedicinal and ecological studies of plants in Garhwal Himalayan in high altitude, where a total of 152 medicinally important plant species were reported, in which 103 were found to be herbs of which some of the species were found to be common with similar use of the present study. A similar study on ethnomedicinal plants of other parts of the country is also done by Joshi et al. [14] in Kumaun Himalaya. Negi et al. [15] collected information of 50 plant species regarding their mode of preparation and use of Raji tribes in Uttarakhand Himalaya where few plants were common but their uses were again also reported differently.

The oral consensus of local inhabitants represents that, in each study site, the majority of inhabitants agreed with the same statement as the information collected on plants. A similar study was carried out by Bhat et al. [11] where the consensus of informants for the roots and rhizomes of plants was the most frequently used (68%). Singh and Rawat [16] also reported that roots are the most used plant parts. According to Keter and Mutiso [17], the leaves are the most frequently used plant parts. However in the present study, the majority consensus on the most used plant part was the root.

## 4. Conclusions

The dialectical relationship between indigenous knowledge and practices shapes the ecosystem and affects the constituent plant population. By incorporating indigenous knowledge and use in the process of scientific research, new hypotheses for the sustainable conservation of resources can be developed. Indigenous knowledge and use have to be analyzed to develop appropriate management measures that build on both scientific and local knowledge. Due to the changing perception of local people and the ever-increasing influence of global commercialization and socioeconomic transformation, indigenous knowledge of plant resource use is constantly diminishing. Due to the lack of organized sustainable and scientifically monitored cultivation and harvesting, lack of proper management techniques, and lack of awareness of social factors, the number of useful plant resources is decreasing at an alarming rate. Furthermore, indigenous knowledge on the use of lesser-known plants is also rapidly declining.

## Figures and Tables

**Table 1:** Ethnobotanically commonly used plant species.

Sl. number	Species name	Voucher number	Common name	Family	Parts used	Ethnobotanical Use
1	*Achillea millefolium *L.	UHF-11713	Chuang	Asteraceae	Leaves	Leaves crushed and used for curing indigestion, cough, cold, and toothache problems.

2	*Aconitum heterophyllum *Wall. ex Royle	UHF-11754	Patrees	Ranunculaceae	Roots	Used as antipyretic and astringent. Roots are ground and mixed with sugar and eaten with water to relieve stomach pain.

3	*Aconitum violaceum *Jacq. Ex Stapt	UHF-11702	Pattees	Ranunculaceae	Roots	A small piece of tuberous roots is given with hot water in cases of fever due to cold.

4	*Aconogonum molle* (D. Don) H. Hara	UHF-11768	Tarodi	Polygonaceae	Leaves, stems	Leaves are cooked as vegetable. Young stems are sour and quench thirst in case of nonavailability of water.

5	*Allium victorialis *L.	UHF-12376	Happu	Amaryllidaceae	Leaves, roots	Leaves are used as vegetable and substitute for garlic. Roots are not harvested so as to allow plants to regrow.

6	*Ainsliaea aptera* DC.	UHF-11703	Sathjalari/Sathjalori/Karvibooti	Asteraceae	Roots	Roots powder is applied on cut and wounds, and also stomachache, diuretic.

7	*Anaphalis nubigena *DC.	UHF-11777	Bhujlu	Asteraceae	Leaves	Fibre collected from backside of leaves is rubbed with runka (iron instrument) to produce fire.

8	*Angelica glauca *Edgew.	UHF-12305	Chora	Apiaceae	Roots	(i) The root is dried and roasted in ghee and powdered. The powder is used as spice. (ii) Root powder with black salt (kala namak) is given in flatulence and dyspepsia. (iii) Dry roots powder mixed with oil is applied to cure oedema.

9	*Arenaria festucoides* Benth.	UHF-12775	Mumri	Caryophyllaceae	Leaves	Considered best fodder for sheep.

10	*Artemisia vulgaris *L.	UHF-12310	Chharmar Hindi: Nagdauna	Asteraceae	Leaves	Fresh juice of leaves cures itching in eyes, occurring during summer months.

11	*Aster himalaicus *C. B. Clarke	UHF-12394	Raktjadi	Asteraceae	Roots	Any person having blood problem during stools is given the decoction of its roots.

12	*Bistorta amplexicaulis *(D. Don) Greene	UHF-11741	Greene	Polygonaceae	Rhizome	Lal chai the coloured rhizome is cut in small pieces.

13	*Cannabis sativa *L.	UHF-11763	Bhang	Cannabaceae	Seed	Seeds powder mixed with oil for typhoid, jaundice, malaria, and fever.

14	*Chaerophyllum reflexum *Lindl.	UHF-12390	Sojuga, bhai	Apiaceae	Roots, seeds	Roots are used for stomach complaints. Seeds infusion used in body pain, cold, and cough.

15	*Dactylorhiza hatagirea *(D. Don) Soó	UHF-11706	Salam panja	Orchidaceae	Roots	Energetic, health tonic, and nervine tonic. Root is eaten in case of headache. Tubers paste applied on cut and wounds.

16	*Datura stramonium *L.	UHF-9888	Dhaintura	Solanaceae	Seed	Seed is dried and ground. The powder of seeds is mixed with mustard oil and boiled. After cooling, it is applied to pained joints to relieve pain.

17	*Dioscorea deltoidea* Wall. Ex Grieseb.	UHF-12383	Khaldri	Dioscoreaceae	Roots	Roots are powdered and put in wooden pot with holes to protect woolen clothes from insect attack.

18	*Foeniculum vulgare *Mill.	UHF-12391	Saunf	Apiaceae	Seed	Used as condiment.

19	*Fragaria vesca *L.	UHF-11712	Bubal	Rosaceae	Fruits, roots	Fruits are edible. Roots infused with ghee butter and honey is used to cure dysentery.

20	*Gentiana kurroo *Royle	UHF-11761	Kadoo	Gentianaceae	Leaves	Leaves are eaten during fever.

21	*Heracleum candicans* Wall. ex DC.	UHF-11711	Patala	Apiaceae	Roots	Root is ground and the paste is used in case of snake bite. Sour lassi is mixed with paste and given to patient.

22	*Impatiens balsamina *L.	UHF-9894	Tilpar	Balsaminaceae	Seed, whole plant	When young, the plant is used for mehandi. It produces very dark colour. Seeds are very tasty.

23	*Meconopsis aculeata* Royle	UHF-11759	Kalkotti	Papaveraceae	Root	Root is ground and given to animals along with salt for creating resistance to diseases.

24	*Mentha longifolia *(Linn.) Huds.	UHF-12393	Pudina	Lamiaceae	Root, leaves	Fresh root is dried, powdered, mixed with pepper, and then given to patient suffering from piles. Leaf extract is used to cure vomiting, dysentery, stomachache, and headache.

25	*Origanum vulgare *L.	UHF-11721	Marua	Lamiaceae	Whole plant	Utensils of milk and ghee are washed using this plant as it gives good aroma to the utensil.

26	*Oxalis corniculata *L.	UHF-11709	Amblu/Malori	Oxalidaceae	Leaves	Shoots are crushed and juice extract is used in boils, cuts, wounds, fever, and dysentery.

27	*Oxyria digyna *(Linn.) Hill	UHF-12340	Chhoti Chukri	Polygonaceae	Leaves	Leaves are very sour and are used as digestive and purgative by making chutney.

28	*Panicum miliaceum *L.	UHF-11704	Chowla	Poaceae	Seed	Seed is edible.

29	*Picrorhiza kurroa *Royle ex Benth.	UHF-12354	Karoo	Plantaginaceae	Roots	Roots powder consumed during stomachache, jaundice, and diarrhea. Chewing of 2-3 leaves acts as antipyretic. Decoction of leaves is sprinkled in field of wheat which prevents insect attack.

30	*Plantago lanceolata *L.	UHF-11748	Isabgol	Plantaginaceae	Husk	Husk is good for some stomach ailments.

31	*Pleurospermum candollei*(DC.) Clarke	UHF-11776	Baandi	Apiaceae	Seed	Seeds are boiled along with tea to escape cold and substitute for fennel.

32	*Podophyllum hexandrum *(Royle) Wedd.	UHF-11716	Bankaakdu	Berberidaceae	Rhizome, fruits, roots	Rhizome used for kidney problem and as health tonic. Fruit is eaten by Gaddis to cure chronic constipation. Roots are ground and mixed with sugar and decoction is given to patient.

33	*Potentilla argyrophylla *Wallich	UHF-11773	Tama	Rosaceae	Leaves	Decoction of leaves is used to treat diarrhea, arthritis, and kidney stones.

34	*Potentilla nepalensis* Hook.	UHF-12389	Dori	Saxifragaceae	Roots	Roots powder is used to cure stomach disorder.

35	*Primula denticulata *Sm.	UHF-12350	Palak/Jalkutral	Ranunculaceae	Leaves	Leaf paste is used for abdomen pain.

36	*Primula floribunda *Wall.	UHF-12386	Baasdu	Primulaceae	Flower	Flowers are believed to have supernatural power to ward off devils and people knowing witchcraft. Flowers increase beauty of hair of ladies.

37	*Prunella vulgaris *L.	UHF-11745	Gudli	Lamiaceae	Stems	Young stems of plants are kept in cluster in living rooms to expel mosquitoes and flies.

38	*Ranunculus laetus* Wall.	UHF-11722	*Bariyara *	Ranunculaceae	Leaves and flower juice	Leaves and flowers juice are used for curing eye diseases.

39	*Rheum australe* D. Don.	UHF-9878	Chuchchi/Rewandchini	Polygonaceae	Roots	Roots and rhizomes paste/powder/infusion/decoction are used in boils, headache, muscles injury, gastric problems, and also as tooth powder.

40	*Rosa moschata *Miller	UHF-9897	Kojai	Rosaceae	Fruits	Fruit is eaten because of its vermicidal properties

41	*Selinum vaginatum* C. B. Clarke	UHF-11756	Bhootkaisi	Apiaceae	Roots	Roots are ground with wheat flour. Seed is also added and then good quality wine is prepared.

42	*Saussurea gossypiphora *D. Don	UHF-11719	Ghuggi	Asteraceae	Flower	Considered very auspicious and kept for worship along with baan and also used in havan and is known to purify air.

43	*Saussurea taraxifolia* Wall.	UHF-9880	Shivjata	Asteraceae	Roots	Little quantity of root is ground and mixed in boiling milk and given to pregnant lady before delivery. This prevents pain and helps in easy delivery. People with falling hair are advised to use root powder for washing hair. Dhuni is also given to ward off evil spirits

44	*Saussurea lappa* (Decne.) Sch. Bip.	UHF-9876	Kuth	Asteraceae	Seed	Oil of the seeds is applied on aching joints to relieve pain.

45	*Sedum ewersii *Ledeb.	UHF-11760	Kirti	Crassulaceae	Whole plant	Whole plant is ground after drying. One teaspoon of powder is mixed with hot milk and given to patient suffering from piles.

46	*Sempervirens sedoides* Decaisne	UHF-11789	Chidi di Pinnadi	Crassulaceae	Leaves	Paste of leaves helps to remove pimples.

47	*Swertia speciosa *D. Don	UHF-11752	Bambiri	Gentianaceae	Roots	Roots are ground with water and put into eyes like surma to relieve snow burnt eyes.

48	*Thymus serpyllum *L.	UHF-11732	Ban-ajwain	Lamiaceae	Whole plant	Flavouring agent is also eaten for stomach ailments.

49	*Urtica dioica *Linn.	UHF-12382	Ain/Bichhu buti	Urticaceae	Roots	Roots are wrapped in black cloth to get rid of ill will. Leaves are boiled in hot water and then cooked as vegetable.

50	*Valeriana jatamansi* Jones	UHF-11789	Nak Nahani	Valerianaceae	Roots	Roots and stems are used for havan (incense).

51	*Verbascum thapsus *Linn.	UHF-11714	Hanuman ra lingna	Scrophulariaceae		Used for havan and scaring evil spirits.

52	*Viburnum cylindricum* Buch.-Ham. ex. D. Don	UHF-12369	Karneh	Sambucaceae	Seeds	Seeds are eaten with water. Good for relieving constipation.

53	*Viola pilosa *Blume	UHF-11726	Banaksha	Violaceae	Flower	Decoction of flowers is used in case of cough and cold.

54	*Viola serpens* Wall. ex. Roxb.	UHF-11743	Napalu	Violaceae	Flower	Decoction of flowers is used in case of cough and cold.
